# Differences in microbial community structure induced by tillage practices under straw return associated with metabolic functions

**DOI:** 10.3389/fmicb.2026.1794771

**Published:** 2026-05-08

**Authors:** Shangjun Su, Haoruo Li, Wenguang Li, Zhenping Yang, Qiang Zhang

**Affiliations:** 1 Soil Health Laboratory in Shanxi Province, Shanxi Agricultural University, Taiyuan, Shanxi, China; 2 Institute of Eco-environment and Industrial Technology, Shanxi Agricultural University, Taiyuan, Shanxi, China; 3 College of Resources and Environment, Shanxi Agricultural University, Taigu, Shanxi, China; 4 College of Agriculture, Shanxi Agricultural University, Taigu, Shanxi, China; 5 Shanxi Provincial Key Laboratory of Crop Ecology and Efficient Water & Nutrient Use, Shanxi Agricultural University, Taigu, Shanxi, China

**Keywords:** dryland winter wheat, soil metabolites, soil microorganisms, straw return, tillage management

## Abstract

This study aimed to elucidate the biological mechanisms through which different tillage practices affect wheat yield and nutrient use efficiency under straw return conditions, focusing on rhizosphere microbial communities, metabolite profiles, and their interactions to inform improved agricultural management. The field experiment tested three treatments: no-tillage with all straw mulching (SN), rotary tillage straw return (SR), and plow tillage straw return (SP). Using high-throughput sequencing and liquid chromatography-tandem mass spectrometry, we investigated how different treatments affected the microbial community in wheat rhizosphere soil and metabolic functions through microbiome and non-targeted metabolomics analyses. SN helped to increase the wheat yield, soil nutrient content in the plow layer, and enzyme activity. Compared with SN, the yields were 8.3% and 12.4% lower under SR and SP, respectively, the soil organic carbon contents were 13.2% and 5.6% lower, and the pH values were 2.7% and 1.2% higher. Tillage practices significantly altered the composition and diversity of the bacterial and fungal communities. The species richness of bacterial and fungal communities followed the order of: SP > SR > SN and SN > SP > SR, respectively. The stabilities of the bacterial and fungal communities exhibited the same distribution pattern. Principal coordinate analysis and PERMANOVA indicated that under straw return, different tillage practices led to significant separation of soil bacterial and fungal communities. Furthermore, Actinobacteria and Proteobacteria contributed most significantly to differences in the bacterial community structures, and Ascomycota and Basidiomycota contributed most significantly to differences in the fungal community structures. Under straw return, different tillage practices significantly altered soil metabolite composition. Bacterial communities correlated more strongly with soil metabolites than fungal communities. Compared with SN, metabolic pathways for different metabolites were enriched under SR and SP, and all were related to amino acid metabolism, putatively mainly in the valine, leucine, and isoleucine biosynthesis pathways. Collectively, our findings demonstrate that tillage practice is a key regulator of straw-amended soil ecosystems, and that no-till with straw mulching optimizes yield and nutrient efficiency primarily by enhancing the functional synergy between the rhizosphere microbiome and metabolome.

## 1 Introduction

Returning straw to agricultural soil is an environmentally friendly measure that is widely recognized for ensuring food security and rehabilitating soil due to its positive impacts on the environment and soil health, such as improving the soil quality, enhancing soil carbon sequestration, maintaining soil moisture, and boosting nutrient cycling (Turmel et al., 2015). However, some studies have shown that straw return (SR) can lead to decreases in N availability and soil temperatures in certain situations, with negative impacts on crop growth (Zhang et al., 2025). According to a study of tillage practices by (Turmel et al. 2015), straw mulching (SN) and burying can lead to significantly different outcomes in terms of the soil nutrient status and crop growth. No-tillage with straw mulching can improve the soil structure by reducing soil disturbance to increase the water and nutrient use efficiency, thereby boosting grain yields (Li et al., 2026; Yuan et al., 2022). However, (Pittelkow et al. 2015) indicated that low seedling rates and uneven seeding caused by no-tillage with straw mulching can decrease crop yields. Cleary, the yield is just one outcome of agriculture, and enhancing the soil quality and delivering additional ecosystem services are also important considerations for managers (Jiao et al., 2022). In addition to productivity, understanding the mechanistic interactions between microorganisms and plant roots may help to enhance the potential contributions of tillage practices to sustainable agricultural intensification. Therefore, it is necessary to obtain insights into how soil microbial communities respond directly and indirectly to agricultural practices.

Previous studies have demonstrated that agricultural techniques can potentially modify soil microbial communities, although most focused on non-rhizosphere environments (Cheng et al., 2022). The rhizosphere microenvironment is crucial for plant growth as complex biochemical interactions occur between the cells in plant roots and soil microbes (Kuzyakov and Razavi, 2019). Plant root systems can adjust to environmental or nutrient levels by releasing chemical signals, influencing specific microorganisms (Kuzyakov and Razavi, 2019). Moreover, the rhizosphere microbial community regulates plant adaptation through the release of chemical signals such as hormones and auxins (Shi et al., 2016). Thus, it is remarkable to consider that microbes in the rhizosphere might control crop growth and soil environments through interactions with specific compounds, although our understanding of these interactions is limited (Chen et al., 2019).

In recent years, rapid advances in metabolomics technologies [e.g., liquid chromatography-tandem mass spectrometry (LC-MS/MS)] have facilitated the identification of low-to-medium molecular weight compounds in the rhizosphere (Cheng et al., 2022; Li et al., 2024). In addition, metabolomics technology can help us understand the changes in soil metabolites and metabolic pathways under different agricultural management measures (Cheng et al., 2022; Li et al., 2024; Wang et al., 2024a,b). Moreover, this technique can be combined with microbiome analysis to obtain potential biomarkers as a powerful tool for understanding external disturbances in biological systems (Wu et al., 2022). Therefore, analyzing microbial community compositions, soil metabolite compositions, and metabolic pathways can help us gain a deeper understanding of the complex biological processes in soil. However, few previous studies have investigated the responses of soil metabolites to various tillage methods with straw incorporation, and their effects on rhizosphere microbial communities are not well understood. Thus, in the present study, we combined amplicon sequencing technology with non-targeted metabolomics analysis to elucidate the effects of different tillage practices on soil microbial community structures and metabolite compositions in dryland wheat fields under straw incorporation. We examined the impacts of rhizosphere metabolites on the structures of bacterial and fungal communities to ultimately support the use of crop straw as a valuable resource and sustainable dryland agriculture on the Loess Plateau in China.

## 2 Materials and methods

### 2.1 Site description

The experiment was initiated during June 2016 in Yuanqu County, southern Shanxi Province, China (111°43.3′E, 35°14.4′N). Before the initiation of this study, the experimental plot had been under long-term conventional cultivation of rainfed wheat by local farmers. The study region has a warm temperate monsoon climate, with a yearly accumulated temperature of 4,880 °C, average annual precipitation of 700 mm, average annual evaporation of 1,220 mm, and average air temperature of 13.8 °C. The medium loam soil at the study site was classified as brown Hongli loess according to the USDA classification system for soils (Li et al., 2024). During 2016, the soil properties in the 0–20 cm soil layer were determined as follows: pH, 7.8; organic carbon, 10.5 g kg^−1^; total nitrogen (TN), 0.9 g kg^−1^; available phosphorus (AP), 12.5 mg kg^−1^; and available potassium, 103.7 mg kg^−1^.

### 2.2 Experimental design

The experiment was conducted using a randomized block design, with three treatments: no-tillage with all straw mulching (SN), where maize straw was shredded into 5–10 cm segments and applied on the field's surface; plow tillage straw return (SP) and rotary tillage straw return (SR), where shredded straw was buried in the soil at depths of 35–40 cm and 25–35 cm using a deep plow machine and rotary machine, respectively. In addition, 150 kg ha^−2^ N fertilizer (urea, 46%), 105 kg ha^−2^ P fertilizer (calcium superphosphate, 16%, P_2_O_5_), and 60 kg ha^−2^ K fertilizer (potassium sulfate, 52%, K_2_O) were applied under each treatment. All three treatments were performed in triplicate, resulting in a total of nine experimental plots. The size of each plot was 25 × 40 m.

Winter wheat sowing was conducted in early October each year and harvesting was completed in mid-June during the following year. Maize was sown immediately after harvesting wheat in the summer. Before planting wheat for the upcoming season, the harvested maize straw was chopped and returned to the field. The winter wheat (*Triticum aestivum* L.) cultivar used in the trial was Yannong 21, which has high resistance to cold conditions during spring. The mechanized wide and narrow row sowing technique was adopted (with a wide row of 25 cm and narrow row of 12 cm), with a sowing depth 5 cm and sowing rate of 150 kg ha^−1^. Irrigation measures were not implemented during the winter wheat–summer maize rotation. Pests and diseases were controlled by spraying pesticides, while weeds were manually removed.

### 2.3 Sampling and measurements

#### 2.3.1 Productivity of winter wheat

At the full grain maturity stage for winter wheat, one randomly selected plot (3 m^2^) within each experimental block was cut to determine the aboveground biomass and grain yield. Plants were then separated into grains, stems and leaves, and glumes, and dried at 65 °C. After digesting the samples using the H_2_SO_4_-H_2_O_2_ method, the concentrations of N and P were determined with a continuous flow analyzer (Auto Analyzer 3, Bran+Luebbe, SEAL Analytical GmbH, Germany) and vanadium molybdate yellow colorimetric method.

#### 2.3.2 Soil sampling

Soil samples (0–20 cm) were collected during the overwintering, jointing, and harvest stages in the 2016–2017, 2017–2018, and 2018–2019 winter wheat seasons. In particular, within each experimental plot, root-zone soil was collected from five randomly selected sites. After removing debris and sieving (2 mm), equal volumes were pooled to prepare a single composite sample per plot. Thus, three composite samples were prepared for each treatment (from three replicate plots). Samples were stored at low temperatures during transport and subdivided in the laboratory, where the first part was air dried to measure the soil nutrient contents and enzyme activity levels, and the other two parts were frozen at −80 °C for extracting nucleic acids and metabolites. Given that 70%−80% of wheat root biomass is concentrated in the topsoil (0–20 cm), soil samples from this layer at the harvest stage of the 2018–2019 growing season were subjected to microbial community analysis (with three replicates) and untargeted metabolomic analysis (with six replicates to enhance robustness).

#### 2.3.3 Soil physicochemical properties

The sucrase (SUC) activity was determined using the 3,5-dinitrosalicylic acid assay. The alkaline phosphatase (AKP) activity was measured based on the phenol concentration produced. The urease (URE) activity was determined by using phenol hypochlorite colorimetry (Guan et al., 1986). The soil organic carbon (SOC) content was analyzed by using the K_2_CrO_7_-H_2_SO_4_ oxidation method (Bao, 2000). The TN content was determined by acid digestion according to the Kjeldahl method. The molybdenum blue method was used to determine AP after extraction with 0.5 M NaHCO_3_. The available nitrogen content was assessed by NaOH hydrolysis. The pH was measured with a pH meter (Bao, 2000).

### 2.4 Soil DNA extraction and amplicon sequencing

#### 2.4.1 DNA extraction and sequencing

Total soil DNA was extracted using an OMEGA Soil DNA Kit (D5625 01; Omega Bio Tek, Norcross, GA, USA). The DNA concentration was measured using a Qubit 4 fluorometer and the DNA quality by 1% agarose gel electrophoresis. Total DNA was sent to Personal Biotechnology Co. Ltd (Shanghai, China) for high-throughput sequencing on the Pacbio platform. We amplified the bacterial 16S rRNA gene and the fungal full-length ITS gene using the primer pairs 27F/1492R and ITS1F/LR3, respectively.

#### 2.4.2 Bioinformatics analysis

The raw tags obtained by FLASH (version 1.2.7) concatenation were rigorously filtered to obtain clean tags, including tag truncation and filtering tags with lengths less than 75% (Magoč and Salzberg, 2011; Bokulich et al., 2013). After removing chimeric sequences, the final effective tags were obtained. Uparse software (version 7.0.1001) was used to cluster all effective tags for all soil samples, and sequences with similarity ≥ 97% were classified in the same operational taxonomic unit (OTU) (Haas et al., 2011; Edgar, 2013). Taxonomic annotations were obtained for each representative sequence by using the SILVA 138 database for bacteria and the UNITE 7.2 database for fungi (Quast et al., 2012).

### 2.5 Metabolomics analysis

#### 2.5.1 Metabolite extraction

Metabolomics analysis involved the following five steps. First, 400 mg of soil sample was accurately weighed in a 4 ml Eppendorf tube, before adding aqueous methanol solution and 1.2 ml of 2-chlorophenylalanine, and vortexing for 60 s. Next, 200 mg of glass beads were added to the mixed sample, before high-speed grinding at 65 Hz for 100 s. The sample was then immediately ultrasonicated at 25 °C for 35 min, before allowing to settle for 40 min, centrifuging at 15,000 rpm, collecting the supernatant, and filtering through a 0.23 μm membrane filter. The resulting filtrate was retained for subsequent analysis (Ponnusamy et al., 2011; Ng et al., 2012).

#### 2.5.2 Liquid chromatography-tandem mass spectrometry (LC-MS/MS) analysis and bioinformatics analysis

The filtrate described above was subjected to LC-MS/MS analysis using a Thermo Ultimate 3000 chromatograph (Thermo Fisher Scientific, USA). Gradient elution was performed with 0.1% formic acid aqueous solution and 0.1% formic acid/acetonitrile solution at a flow rate of 0.25 ml/min. MS analysis was conducted using electrospray ionization (Thermo Q Exactive Focus), with positive/negative ion spray voltages of 3.50 kV/2.50 kV, sheath gas at 30 arb, and auxiliary gas at 10 arb. The capillary temperature was set to 325 °C, and a full scan was conducted at a resolution of 70,000, spanning a range from 81 to 1,000. Secondary fragmentation utilized higher-energy collisional dissociation (HCD) with a collision energy of 30 eV (Ponnusamy et al., 2011; Sangster et al., 2006).

The dynamic exclusion technique was used to remove interfering signals from the spectrum. Peak identification, filtering, and alignment were conducted using the “XCMS” package in R. Finally, adhering to the Metabolomics Standards Initiative (MSI) convention, we utilized the Human Metabolome Database (HMDB), METLIN, and MassBank, which provided detailed annotations of the MS/MS fragment data for metabolite identification (accurate mass of < 15 ppm and/or match of major MS/MS fragments) (Chan et al., 2011).

### 2.6 Statistical analyses

Analysis of variance was conducted to determine significant differences in each index using SPSS 26 software (SPSS Inc., Chicago, IL, USA), and data were visualized with Origin 2024 (Origin Lab Corp., MA, USA). The response ratios (RRs) for soil nutrients and enzyme activities under SN and SP compared with SR were calculated using the following formula (Zhong et al., 2023):


$$\begin{array}{l} RRs=\ln\;\left(\frac{Xt}{Xc}\right)=\ln\;\left(Xt\right)-\ln\;\left(Xc\right) \end{array}$$

where Xt denotes SN or SP and *Xc* denotes SR.

Principal coordinate analysis (PCoA) was conducted based on the Bray–Curtis distance matrix to evaluate changes in microbial community structures, and visualized using the “microeco” package in R. The alpha diversity indexes were calculated for microbial communities using the “vegan” package in R, permutational multivariate analysis of variance (PERMANOVA), and percent similarity analysis (indicating species contribution rates). The average variation degree (AVD) index was used to evaluate the overall stability of bacterial and fungal communities. The “mixOmics” package in R was utilized to perform partial least squares discriminant analysis (PLS-DA) to assess the metabolite compositions under different treatments. Mantel tests were performed using the “vegan” package to examine the relationships between microbial communities and different metabolite components, based on Bray-Curtis distance for species and Euclidean distance for metabolites. Differential metabolite pathway enrichment analysis was performed using the online tool MetaboAnalyst 6.0 (https://www.metaboanalyst.ca). *P*-value adjustments for multiple comparisons were performed using the false discovery rate (FDR) correction. The “ggvolcano” package in R was used to generate a volcano plot to identify differential metabolites among different treatments. The “pheatmap” package in R was utilized to produce a heatmap to examine the links between differential metabolites and microbial communities.

## 3 Results

### 3.1 Responses of wheat yield and soil properties to different tillage measures under straw return

**Figure 1:**
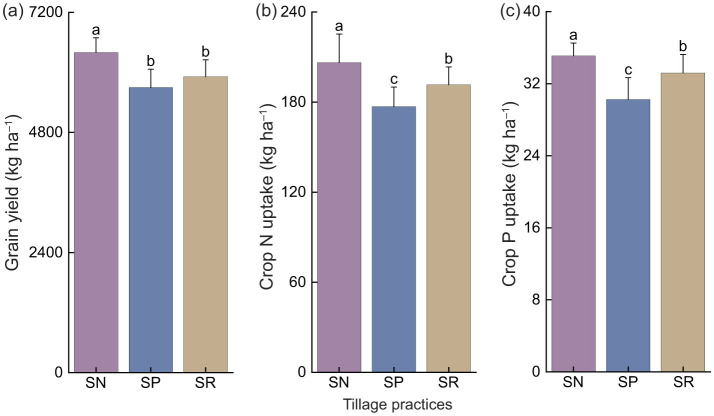
Effects of different tillage practices under straw return on average annual wheat yield **(a)** and plant uptake amounts of nitrogen **(b)** and phosphorus **(c)** (2017–2019, *n* = 9).

**Figure 2:**
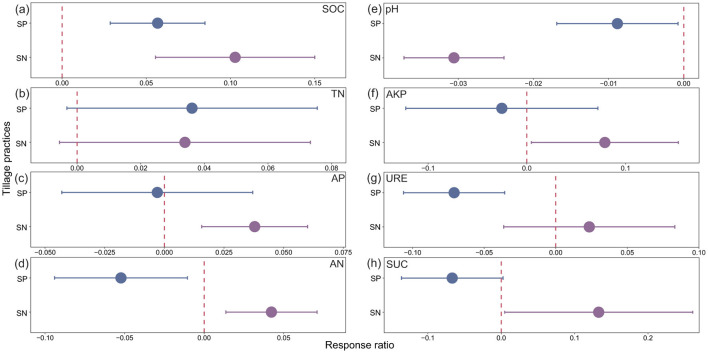
Effects of different tillage practices under straw return on soil nutrients and enzyme activities. The response ratio for each indicator is integrated based on three growth stages (2016–2019). The error bars represent 95% confidence intervals. Error bars that do not overlap with the zero line indicate statistically significant differences (*P* < 0.05). Soil organic carbon (SOC) **(a)**; total nitrogen (TN) **(b)**; available phosphorus (AP) **(c)**; available nitrogen (AN) **(d)**; pH **(e)**; alkaline phosphatase (AKP) **(f)**; urease (URE) **(g)**; and sucrase (SUC) **(h)**.

Different tillage measures under straw return had significant impacts on the growth of winter wheat and soil properties. Compared with SR and SP, the annual wheat yield under SN was 8.2% and 12.3% higher, respectively, phosphorus uptake was 7.7% and 16.6% higher, and nitrogen uptake was 5.7% and 16.1% higher (Figure 1). Comprehensive analysis of the results (Figure 2) showed that both SP and SN improved SOC and TN in the 0–20 cm soil layer compared with SR (0 line), where the SOC level was 13.3% and 5.7% higher under SN than SR and SP, respectively. The relative reduction rates of AP, available nitrogen, AKP, URE, and SUC followed the order of: SP < SR < SN. It should be noted that SP and SN significantly decreased the relative reduction rate for pH compared with SR, but the greatest decrease occurred under SN (Figure 2e), where the pH value was 2.8% and 1.1% lower than those under SR and SP, respectively. Overall, straw return under no-tillage had greater effects on improving the wheat yield and soil nutrient contents compared with straw return under deep plowing and rotary tillage.

### 3.2 Responses of microbial community structures to different tillage measures under straw return

**Figure 3:**
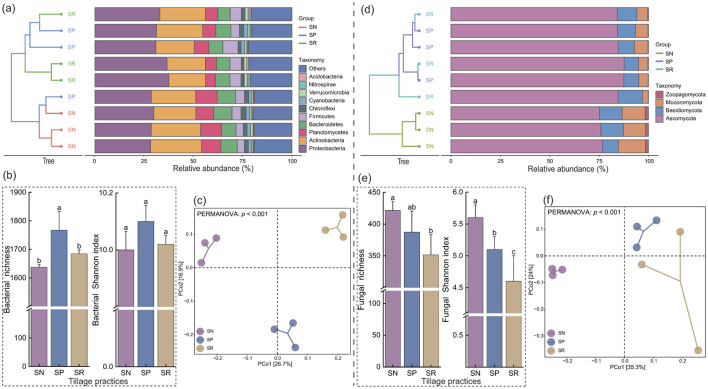
Effects of different tillage practices under straw return on composition **(a, d)** and diversity of soil microbial communities **(b, c, e, f)**.

After removing redundancy and denoising, we obtained 1,246,011 and 1,428,395 high-quality bacterial and fungal sequences, respectively. These sequences were classified into 5,580 bacterial OTUs and 1,516 fungal OTUs. The top five bacterial phyla with the highest average relative abundances were Proteobacteria (32.3%), Actinobacteria (22.1%), Planctomycetes (7.4%), Bacteroidetes (6.9%), and Firmicutes (5.2%; Figure 3a). The dominant fungal phyla were Ascomycota (76.3%), Basidiomycota (16.2%), Mucoromycota (7.0%), and Zoopagomycota (0.5%; Figure 3d). The species composition clustering results indicated that both bacterial and fungal communities were mostly clustered separately, where those under SN alone clustered together, and those under SP and SR clustered together (Figures 3a, d). The species richness results showed that the species richness in bacterial communities followed the order of: SP > SR > SN (Figure 3b); and the species richness in fungal communities followed the order of: SN > SP > SR (Figure 3e). The PCoA results indicated that different tillage measures under straw return led to significant separation of soil bacterial and fungal communities (Figures 3c, f). PERMANOVA also verified the significant responses of the microbial community structures to different tillage measures.

**Figure 4:**
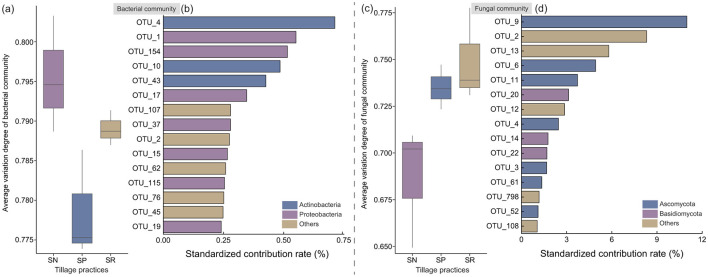
Effects of different tillage practices under straw return on average variation degree (AVD) **(a, c)** and species contribution rate **(b, d)**.

The AVD indexes for bacterial and fungal communities (a lower index value indicates higher community stability; Figures 4a, b) exhibited the opposite distribution pattern to the species richness (Figures 3b, e), showing that no-tillage under straw return could decrease the stability of bacterial communities, making them more susceptible to environmental disturbances. However, no-tillage significantly altered the species abundances in fungal communities, whereas the species compositions remained relatively stable. Thus, substantial disparities were found in the structures of the bacterial and fungal communities under different treatments. SIMPER analysis showed that the phyla with the greatest contributions to the structural differences in bacterial communities were Actinobacteria and Proteobacteria, and the phyla with the greatest contributions to the structural differences in fungal communities were Ascomycota and Basidiomycota (Figures 4c, d), where OTU_4 and OTU_9 were the species that contributed most, respectively.

### 3.3 Responses of soil metabolite compositions to different tillage measures under straw return

**Figure 5:**
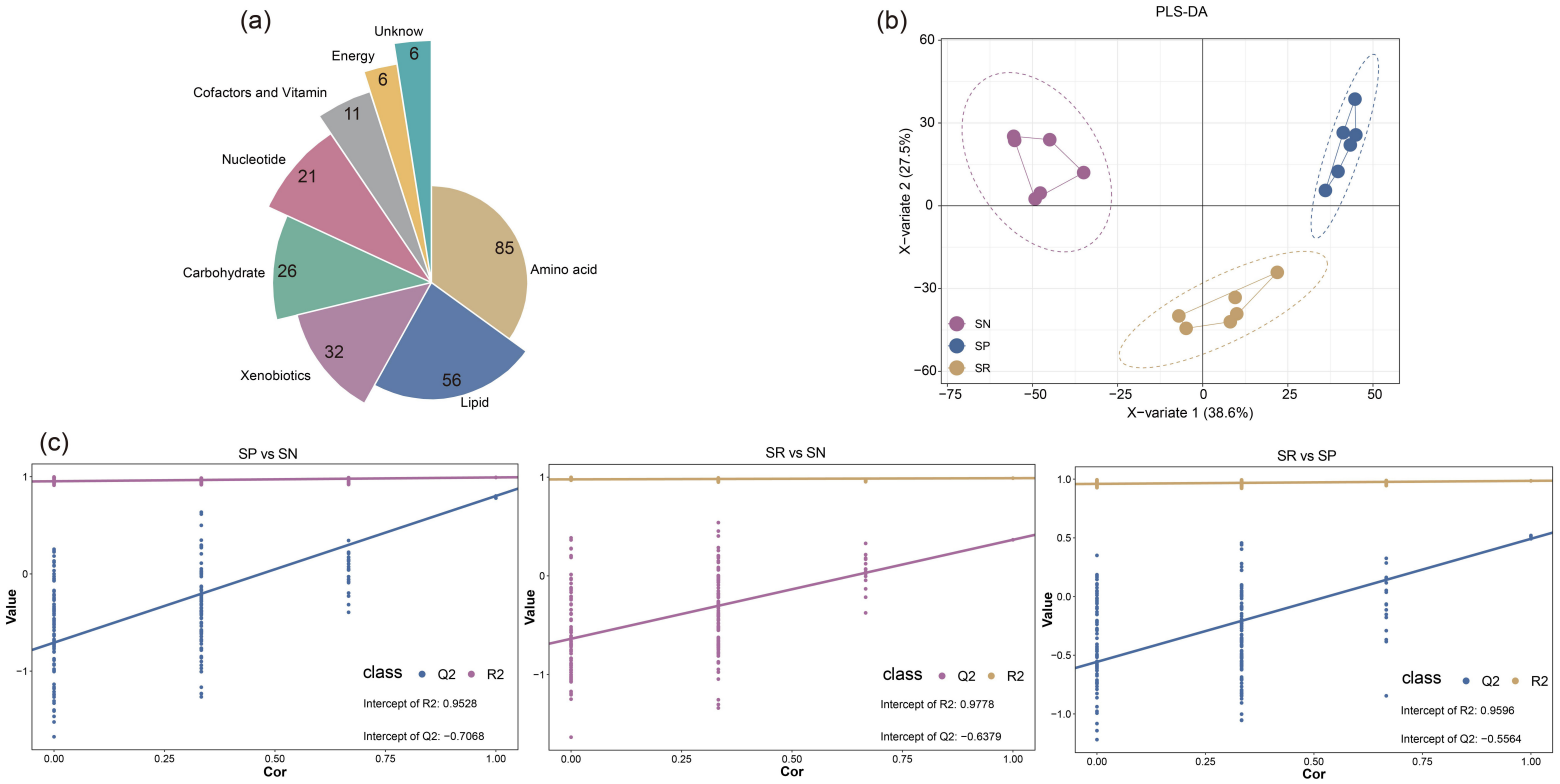
Effects of different tillage practices under straw return on composition **(a, c)** and classification **(b)** of soil rhizosphere metabolites.

**Figure 6:**
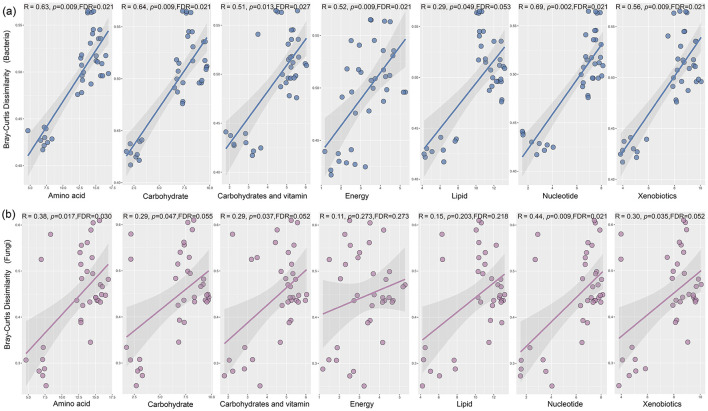
Relationships between soil metabolites and bacterial **(a)** and fungal **(b)** community compositions.

Soil metabolites were extracted and analyzed using LC-MS to investigate how rhizosphere soil metabolites responded to different tillage measures with straw return and their interactions with bacterial and fungal communities. In total, 243 soil metabolites were detected across all of the samples (Figure 5a). According to their molecular structures, these metabolites were categorized as 85 amino acids, 56 lipids, 32 exogenous substances, 26 carbohydrates, 21 nucleotides, 11 cofactors and vitamins, six energy substances, and six unidentified metabolites. PLS-DA showed that the different tillage practices combined with straw return obtained clearly different soil metabolite profiles, with distinct separation under SN, SP, and SR (Figure 5b). In addition, the PERMANOVA test results showed that the PLS-DA model had high explanatory power (*R*^2^ close to 1) and predictive ability (*Q*^2^ close to 1; Figure 5c). Moreover, the Mantel test revealed that although significant positive correlations existed with bacteria for nearly all metabolite classes (except lipids; Figure 6a), only amino acids and nucleotides were significantly correlated with fungi (Figure 6b).

### 3.4 Identification of differential metabolites

**Figure 7:**
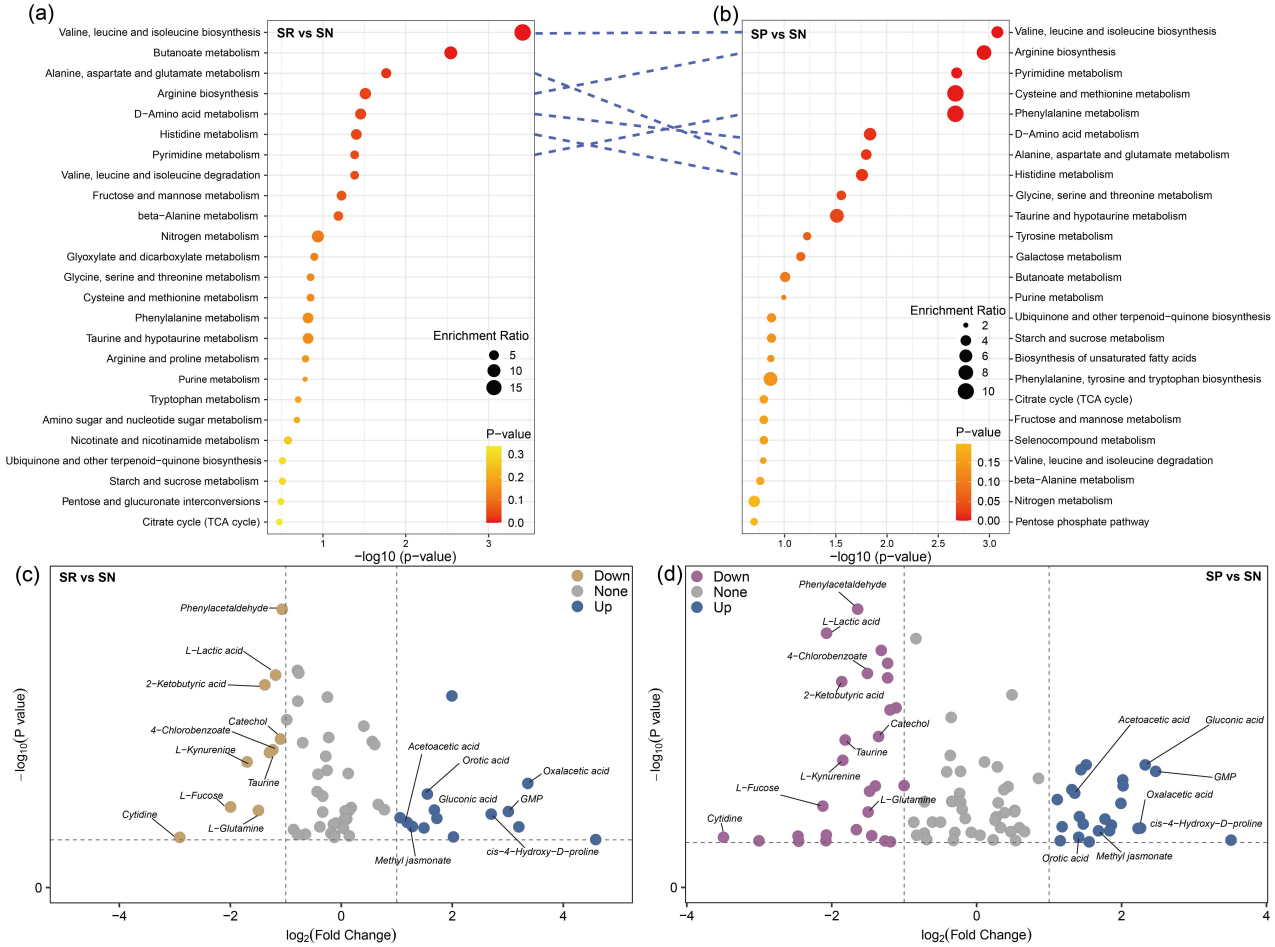
Identification of enrichment analysis **(a, b)** and differential metabolites **(c, d)**.

Analysis of differential metabolites using KEGG pathway enrichment revealed that compared with SN, SR (Figure 7a) and SP (Figure 7b) enriched differential metabolites in six of the top eight metabolic pathways, which were all related to amino acid metabolism. These pathways included the biosynthesis of valine, leucine, and isoleucine, arginine biosynthesis, phenylalanine metabolism, D-amino acid metabolism, metabolism of alanine, aspartic acid, and glutamic acid, and histidine metabolism. In particular, the metabolites putatively enriched in the valine, leucine, and isoleucine biosynthesis pathway appeared to be most abundant under all treatments. These results further confirmed that the metabolic functions may have been similar under SR and SP, whereas SN could have induced specific changes in these metabolic functions.

**Figure 8:**
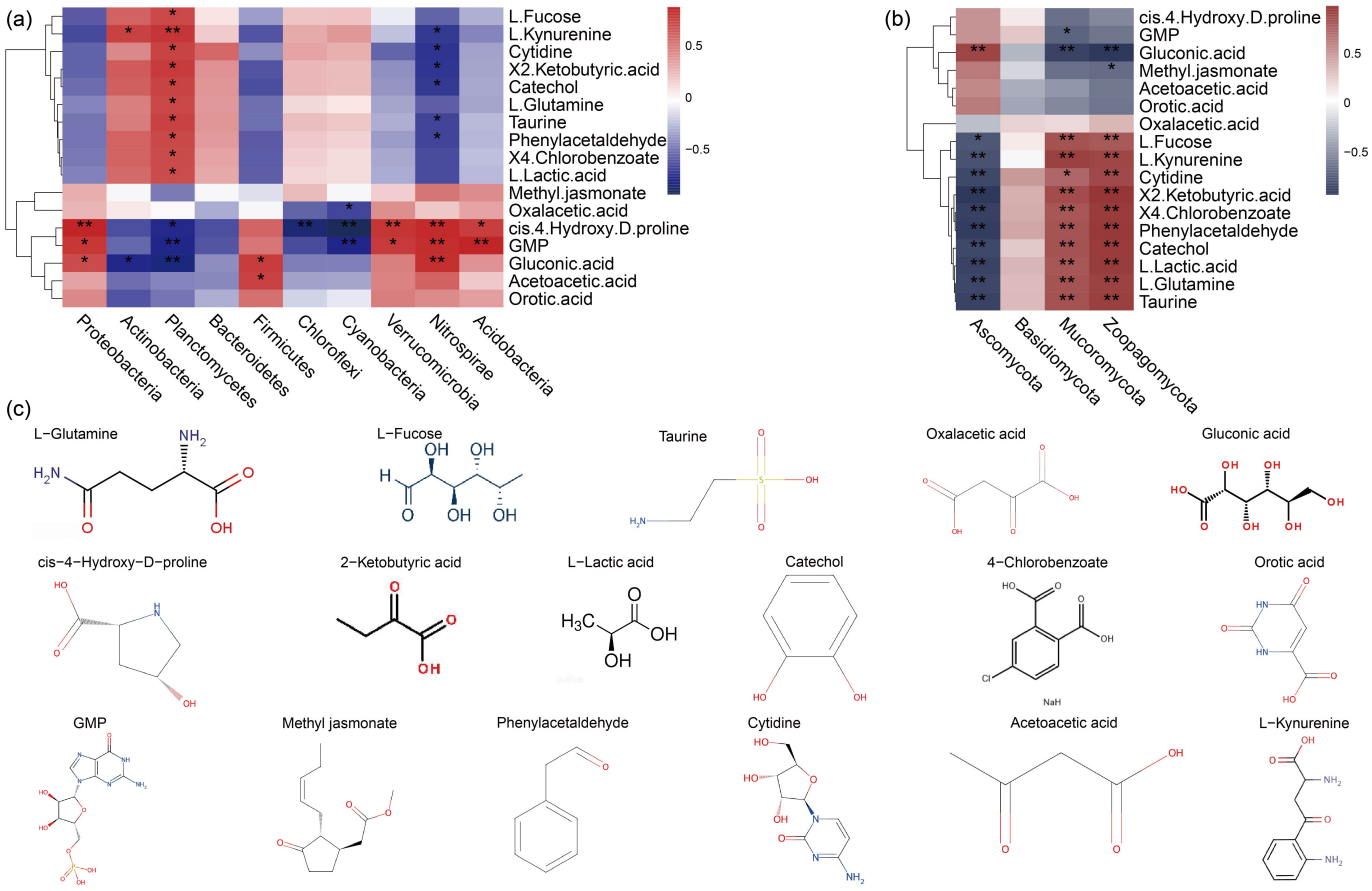
Relationships between differential metabolites and bacterial **(a)** as well as fungal **(b)** species at the phylum level, and structural formulae for differential metabolites **(c)**.

Volcano plots were utilized to further screen for differential metabolites. Compared with SR and SP, SN led to the upregulation of acetoacetic acid (C_4_H_6_O_3_), orotic acid (C_5_H_4_N_2_O_4_), oxalacetic acid (C_4_H_4_O_5_), gluconic acid (C_6_H_12_O_7_), GMP (C_10_H_14_N_5_O_8_P), methyl jasmonate (C_13_H_20_O_3_), and cis-4-hydroxy-D-proline (C_5_H_9_NO_3_; Figures 7c, 8c), as well as the downregulation of phenylacetaldehyde (C_8_H_8_O), L-lactic acid (C_3_H_6_O_3_), 2-ketobutyric acid (C_4_H_6_O_3_), catechol (C_6_H_6_O_2_), 4-chlorobenzoate (C_5_H_9_NO_3_), L-kynurenine (C_10_H_12_N_2_O_3_), taurine (C_2_H_7_NO_3_S), L-fucose (C_6_H_12_O_5_), cytidine (C_9_HN_3_O_5_), and L-glutamine (C_5_H_10_N_2_O_3_; Figures 7d, 8c).

The correlations between the top 10 species (at the phylum level) in terms of their relative abundances and differential metabolites indicated significant associations between bacteria in Planctomycetes and Nitrospirae, as well as fungi in Ascomycota, Mucoromycota, and Zoopagomycota, and most metabolites. Thus, these species may have contributed to the production of most of the soil metabolites (Figures 8a, b). Moreover, the metabolites comprising cis-4-hydroxy-D-proline and GMP had close relationships with bacteria but weaker associations with fungal species.

## 4 Discussion

### 4.1 Responses of wheat yield and soil nutrients to different tillage practices under straw return

In the present study, we found that the yield and nutrient uptake were highest under SN, followed by SR and SP (Figure 1). Many previous studies conducted on the Loess Plateau and on a global scale have demonstrated that no-tillage combined with straw return can yield higher crop outputs than traditional tillage methods, but the effect on increasing yields does not apply to all regions and crops (Yang et al., 2020; Achankeng and Cornelis, 2023; Liang et al., 2023). It is widely acknowledged that no-tillage with straw mulching enhances crop growth by enhancing the physical, chemical, and biological properties of the soil. Returning straw to the field effectively reduces soil moisture losses through evaporation, inter-plant transpiration, and evapotranspiration ratios, as well as increasing pre-planting soil moisture retention and providing some resistance to wind and water erosion, thereby significantly enhancing the soil moisture content and water use efficiency (Wang et al., 2018). It is possible that straw mulching reduces damage to soil aggregates, thereby improving the aggregate structure (Liang et al., 2021; Jiang et al., 2018). The improvement obtained in the soil water retention performance by protective tillage practices, such as no-tillage combined with straw mulching, will promote the decomposition of organic materials, increase the soil SOC content, promote soil particle cementation, increase aggregate formation, improve the soil water, gas, and heat conditions, and achieve increases in crop yields (Zhao et al., 2023).

In addition to the yield, the SOC content was significantly higher under SN than SP and SR (Figure 2a), which could be explained as follows: (i) no-tillage may modify the soil water dynamics, lower the soil permeability, and influence aerobic microbial communities by decreasing their stimulation (Song et al., 2022); (ii) by minimizing soil disturbance, no-tillage can promote the formation of large aggregates that serve as barriers, restricting the access of decomposers to organic materials; and (iii) no-tillage can lead to increases in iron and aluminum oxide levels, which serve as key binding agents, promoting soil aggregate formation through their potent flocculation effect, thereby boosting the physical protection of SOC (Xu et al., 2020). Overall, no-tillage leads to higher SOC stability and a lower mineralization rate. We also found that SN clearly reduced the soil pH compared with SP and SR (Figure 2e), possibly because under conditions where the crop straw was in undisturbed soil, the decomposition of organic matter increased the H^+^ concentration and decreased the concentrations of alkaline cations, such as Ca^2+^ and Mg^2+^, to decrease the pH level and affect the alteration of AP in the soil (Zhao et al., 2022).

### 4.2 Responses of rhizosphere microbial community structures to different tillage practices under straw return

In the present study, the species richness of bacterial communities was significantly lower under SN compared with SP and SR, but the species richness of fungal communities was significantly higher, and thus the bacterial community stability was lower and the fungal community stability was higher under SN (Figures 3b, e). These changes may have been related to differences in the sensitivities of the soil bacterial and fungal communities to environmental changes. Typically, soil bacteria are small with a high turnover rate, enabling them to respond rapidly to environmental changes such as fertilization, moisture levels, and pH. The increased nitrogen input from straw incorporation may enhance the leaching of nitrate and base cations. The dissociation of weakly acidic compounds derived from straw (i.e., H_2_CO_3_ and organic acids) is the primary cause of alkaline leaching, leading to a reduction in pH, which can significantly impact the diversity, composition, and functions of soil bacteria (Lyu et al., 2025). Similarly, in the present study, the organic carbon and available nitrogen contents were higher under SN and the pH was lower (Figure 2).

Conservation tillage has a less disturbing effect on fungal communities, and is beneficial for maintaining microhabitats in agricultural ecosystems (Li et al., 2020). Compared with bacterial communities, fungal communities are more vulnerable to physical disruption, which is related to the growth and structure of their hyphae. By linking the straw surface to the soil, fungal hyphae facilitate the comprehensive use of nutrients that are spatially divided. Straw cover also provides benefits to fungi (Zhang et al., 2012; Li et al., 2020; Wang et al., 2020). In addition, covering crops significantly increases the phylogenetic diversity of fungi. According to the insurance hypothesis, a high microbial diversity and community structure can resist declines in their functions because high species diversity provides greater protection. Thus, even if microbial functions are lost, some microorganisms can still maintain their functions, and this ecological buffering mechanism enables them to play the role of a “functional insurance pool” in severe environmental fluctuations, ensuring overall stability of the community by maintaining key metabolic pathways (Li et al., 2020; Lynch and Neufeld, 2015).

In the present study, we found that different tillage methods with straw return substantially changed the β-diversity of bacterial and fungal communities (Figures 3c, f), and this adaptive restructuring of rhizosphere microorganisms may have been related to rhizosphere effects mediated by root exudates (Fan et al., 2023). Soil microbes react strongly to the chemicals secreted by plant roots and shifts in root zone nutrients (Fan et al., 2023). In particular, plant roots produce compounds such as antibiotics and insecticides to repel pathogens and invaders (el Zahar Haichar et al., 2014). Moreover, the metabolites secreted by plant roots can serve as carbon sources and promote the growth of beneficial microorganisms that assist plants with disease control by secreting antibacterial substances (Fan et al., 2025). This process referred to as plant–soil feedback is an important driving force in plant succession and community structure development. Establishing a connection between these compounds and the composition of the microbial community can help elucidate the mechanisms responsible for the formation of microbial communities in rhizosphere soil.

### 4.3 Composition of soil metabolites and relationship with microbial communities

Root exudates are crucial for shaping the composition and progression of microbial communities. In the present study, we demonstrated that different tillage practices combined with straw return modified the composition of metabolites in the rhizosphere, especially amino acids and lipids, with lesser effects on exogenous substances and carbohydrates (Figures 5a, b). In addition, the differential metabolites between SN and SR, as well as the differential metabolites between SN and SP, were mainly enriched in the same metabolic pathways, and mostly related to amino acid metabolism (Figures 7a, b). External carbon and nitrogen inputs can stimulate microbial assimilation and differentiation, thereby affecting the abundances of amino acids. The main sources of free amino acids in soil are microbial degradation of proteins, plant root exudates, and animal metabolites (Liu et al., 2023). Amino acids are important soil organic nitrogen components that can be absorbed, transported, and metabolized by plant roots, and their synthesis and mineralization greatly affect the storage and stability of SOC. However, soil microbes have a greater capacity for the absorption of free amino acids than plant roots, highlighting their role as major competitors in amino acid absorption (Czaban et al., 2018).

We also found significant positive correlations between amino acids and the β diversities of bacterial and fungal communities (Figure 6). Amino acids play crucial roles in nitrogen transformation and bioenergy supply as core intermediates in the soil nitrogen cycle and important sources of nutrients. Thus, amino acids are carbon and nitrogen sources in microbial respiration and metabolic processes, but also crucial for the formation of microbial communities (Liu et al., 2023). Therefore, amino acid metabolism in the soil reflects the overall activity of microorganisms and is closely related to the accumulation of soil nutrients and improvements in their levels (Geisseler et al., 2010). In addition, amino acids are decomposed into ammonia through the action of ammonifying bacteria, and nitrifying bacteria then convert ammonium nitrogen into nitrate nitrogen, thereby transforming organic nitrogen into inorganic nitrogen (Li et al., 2024). Moreover, amino acids act as signaling molecules between soil microbes and host plants, potentially promoting the growth of some microorganisms but suppressing that of others (Guo et al., 2021).

Compared with SR and SP, the differential metabolites under SN were putatively mainly enriched in the biosynthetic metabolic pathways for valine, leucine, and isoleucine (Figures 7a, b), which may have been related to the significant decrease in the soil pH under no-tillage straw mulching (Figure 2e). Returning straw to the field reduces the soil bulk density, but also inhibits water evaporation in high salt soils to ultimately effectively reduce the soil salt concentration (Song et al., 2025). Amino acids are key structural and metabolic compounds in plants, with essential roles in salt tolerance by plants. A significant change in the environmental pH greatly affects the contents of amino acids such as valine, leucine, and isoleucine, and the effect on the balance between ions and reactive oxygen species enhances salt tolerance by plants, acting as an osmotic regulator to protect plants from salt damage (Wang et al., 2016). Amino acids also serve as precursors for many signaling molecules, which can enhance salt tolerance in plants (Shen et al., 2025). For example, the rapamycin signaling target in plants is activated by 15 amino acids and regulated by leucine. Therefore, the metabolic and functional associations between leucine, isoleucine, and valine are strong, and they jointly play roles in regulating biological signaling in salt tolerance by plants (Song et al., 2025).

### 4.4 Limitations and prospects

This study employed an integrated microbiome and non-targeted metabolomics approach to assess the overall impacts of three common straw-return regimes, including no-tillage with straw mulching, rotary tillage with straw return, and plow tillage with straw return, on rhizosphere processes and crop productivity in a dryland wheat system of the Loess Plateau. However, as all treatments involved straw return and no straw-removed tillage control was included, the individual contributions of tillage intensity, straw placement, and their associated micro-environment and decomposition dynamics remain conflated. This design limitation restricts clear mechanistic interpretation of the observed effects. Future work should implement a factorial experiment incorporating straw-removed treatments to disentangle the independent and interactive effects of tillage and straw management on rhizosphere functioning. Furthermore, targeted MS/MS quantification of key metabolites within the putatively enriched pathways, such as valine, leucine, isoleucine, and their precursors, would be a valuable focus for future validation studies to confirm and quantify these metabolic changes.

## 5 Conclusion

In the present study, compared with SR and SP, no-tillage with straw mulching (SN) enhanced the wheat yield, soil nutrient content in the plow layer, and nutrient uptake by dryland wheat on the Loess Plateau. In addition, SN markedly altered the bacterial and fungal community structures in the rhizosphere soil, and enhanced the stability of the fungal community. Moreover, SN significantly altered the soil metabolic profile compared with SR and SP, suggesting a disruption of metabolic pathways involving differential metabolites (particularly those putatively involved in the biosynthesis of valine, leucine, and isoleucine). Both the bacterial and fungal communities had close associations with the metabolite composition, indicating that differences in the rhizosphere microbial community structure caused by various tillage methods were associated with the soil metabolite composition. Thus, non-targeted metabolomics analysis can be an effective tool for evaluating the structure of soil microbial communities. However, various soil metabolites participate in many biochemical processes, and the exact mechanisms that allow them to interact with microbial communities require further investigation.

## Data Availability

The original contributions presented in the study are included in the article/supplementary material, further inquiries can be directed to the corresponding author/s.
